# Glutamate as a non-conventional substrate for high production of the recombinant protein in *Escherichia coli*

**DOI:** 10.3389/fmicb.2022.991963

**Published:** 2022-09-14

**Authors:** Chung-Jen Chiang, Mu-Chen Hu, Thanh Ta, Yun-Peng Chao

**Affiliations:** ^1^Department of Medical Laboratory Science and Biotechnology, China Medical University, Taichung, Taiwan; ^2^Department of Chemical Engineering, Feng Chia University, Taichung, Taiwan; ^3^Department of Medical Research, China Medical University Hospital, Taichung, Taiwan; ^4^Department of Food Nutrition and Health Biotechnology, Asia University, Taichung, Taiwan

**Keywords:** recombinant proteins, glutamate, metabolic engineering, enzyme biorefinery, protein waste

## Abstract

The economic viability of the biomass-based biorefinery is readily acknowledged by implementation of a cascade process that produces value-added products such as enzymes prior to biofuels. Proteins from the waste stream of biorefinery processes generally contain glutamate (Glu) in abundance. Accordingly, this study was initiated to explore the potential of Glu for production of recombinant proteins in *Escherichia coli*. The approach was first adopted by expression of D-hydantoinase (HDT) in commercially-available BL21(DE3) strain. Equipped with the mutant *gltS* (gltS*), the strain grown on Glu produced the maximum HDT as compared to the counterpart on glucose, glycerol, or acetate. The Glu-based production scheme was subsequently reprogrammed based on the L-arabinose-regulated T7 expression system. The strain with gltS* was further engineered by rewiring metabolic pathways. With low ammonium, the resulting strain produced 1.63-fold more HDT. The result indicates that Glu can serve as a carbon and nitrogen source. Overall, our proposed approach may open up a new avenue for the enzyme biorefinery platform based on Glu.

## Introduction

To mitigate global climate change, the biorefinery platform has been implemented to largely produce fuels and chemicals for replacement of counterparts derived from fossil fuels (Saini et al., 2017; Takkellapati et al., 2018; Francois et al., 2020; Sun et al., 2021). The platform technology that manages the sustainable feedstock such as lignocellulosic biomass has many potential applications in industry. The exploitation of microbes for production of lignocellulosic ethanol (LE) presents to be a good example (Liu et al., 2019). However, the development progress of LE is afflicted by its higher production cost as compared to the petroleum price (Tuck et al., 2012). A cascade process has been suggested to produce value-added products prior to biofuels, which renders the biomass-based biorefinery economically viable (Escamilla-Alvarado et al., 2017). Enzymes, functional proteins, organic chemicals, and antibiotics are bio-based products of industrial importance. Among them, enzymes are extensively applied in the sector of biofuels, chemicals, cosmetics, detergent, food, pharmaceuticals, pulp, and textiles (Li et al., 2012; Jegannathan and Nielsen, 2013). Their global market value now reaches billions of dollars while the market volume continuously increases with industrialization.

Recognized as a biotechnology-friendly microbe, *Escherichia coli* has been commonly applied for the mass production of recombinant proteins. Glucose serves as the main carbon source in the culture medium. The dissimilation of glucose proceeding through glycolysis is highly efficient in *E. coli*. However, the presence of excessive glucose results in acetate overflow which handicaps cellular activities (Millard et al., 2021). This issue has been addressed by the strategy of lowering glucose uptake in cells, involving augmentation of the anaplerotic reaction, recruitment of inferior permeases for glucose transport, or inactivation of certain global regulators (March et al., 2002; Perrenoud and Sauer, 2005; Anda et al., 2006). An alternative method aims to improve activity of the tricarboxylic acid (TCA) cycle by decoupling the *arcA*/*arcB*-mediated control circuit (Vemuri et al., 2006). Results of these engineering approaches are generally encouraging. Nevertheless, it remains challenging to overproduce recombinant proteins in cells. The biosynthesis capacity of cells is usually overloaded by the forced expression of a recombinant protein, which leads to the adverse response known as “metabolic burden” (Oh and Liao, 2000). The shortage of cellular resources further triggers the global stress response that disables cell activities (Gill et al., 2000).

The problem of metabolic burden caused by protein overproduction can be partially solved in *E. coli* cultured with rich media (Oh and Liao, 2000). Yeast extract which consists of amino acids, vitamins, and trace growth elements is commonly supplemented as a major component in rich media (Bekatorou et al., 2006). However, its composition varies from batch to batch. This, in turn, renders the culturing condition and productivity of *E. coli* difficult to control (Potvin et al., 1997). Notice that a prerequisite for the LE production calls on the enzymatic hydrolysis of lignocellulose into fermentable sugars. Indeed, there is 20–30% of the total production cost for LE coming from hydrolytic enzymes (Singh et al., 2019). As illustrated with a hydrolytic enzyme (*β*-glucosidase), the techno-economic analysis reveals that glucose mainly contributes to the cost of raw materials that account for 25% of the production cost for the enzyme (Ferreira et al., 2018). Taken together, it is necessary to make more efforts toward achieving the efficient and cost-effective production of enzymes in the biorefinery sector.

In biorefinery, the processing of feedstocks which comprise starch, hemicelluloses, and oil generally leads to the associated production of proteins in the waste stream (Li et al., 2018). The estimated production of protein wastes can reach 100 million tons per year if biomass-derived fuels provide 10% of transportation fuels for the global need (Tuck et al., 2012). Therefore, it is appealing to valorize the current biorefinery process into which the protein-based production platform is integrated (Tuck et al., 2012; Li et al., 2018). Protein waste coming from various sources contains glutamate (Glu) in abundance (Lammens et al., 2012). Taking sugar beet vinasse as an example, the mass fraction of Glu reaches 63% of the total amino acids in the protein content (Deike and Mol, 1996). This apparently makes Glu promising for industrial applications owing to its renewable and sustainable nature. In this context, our previous study illustrated that Glu substituted for yeast extract in the minimal medium was useful for the enzyme production in *E. coli* (Chiang et al., 2020b). In contrast to complex media, minimal media which contain defined nutrient compositions are favorable for scale-up because of their easy preparation and consistent quality. In addition, evolved *E. coli* gains an excellent capability of metabolizing Glu (Chiang et al., 2021b). This study was therefore initiated to explore Glu as a potential substrate applied for the enzyme production. As a result, the recombinant D-hydantoinase (HDT) was highly expressed in the engineered *E. coli* grown on the minimal medium containing Glu that served as a carbon and nitrogen source.

## Materials and methods

### Genetic manipulation

PCR was applied for cloning of the gltS* (mutant *gltS*) gene of BL21(mut) strain with primers (5′-ccatgattacgccaagcttgggctcatcaccaaaaatatg and 5′-gtaacactggcagagcattacaagacggtaaatcagttc). Meanwhile, the vector backbone of pTH19Kr plasmid was produced by PCR with primers (5′-caagcttggcgtaatcatgg and 5′-taatgctctgccagtgttac). Two PCR DNAs were then spliced together by using the ZFusion kit (ZGene Biotech Inc., Taiwan), leading to pTH-gltS* plasmid. pPhi-TrHDTCh is a conditionally-replicating plasmid and carries the HDT gene under the control of the T7 promoter (P_T7_::HDT) (Chiang et al., 2020a). It was implemented to integrate the DNA containing P_T7_::HDT into the bacterial genome at the Φ80 *attB* according to the reported protocol (Chiang et al., 2008). Furthermore, *zwf* and *pgl* were fused with the λP_L_ promoter (PλP_L_). This was conducted by the PCR-amplification of the patch DNA from either pPR-zwf or pSPL-pgl plasmid with primers RC11417/RC11418 or RC13034/RC13035 (Saini et al., 2016). The patch DNA mainly comprised the LE*-kan-RE*-PλP_L_ cassette flanked by homologous extensions. The genomic insertion of the DNA cassette proceeded through the λ Red-mediated homologous recombination after the patch DNA was electroporated into the host cell.

### Protein production

LB medium consisting of 10 g/l tryptone, 5 g/l yeast extract, and 5 g/l NaCl was routinely used to prepare bacterial strains for overnight. The plasmid-carrying strain was maintained in the presence of 30 μg/ml ampicillin (Amp) or 50 μg/ml kanamycin (Kan). The overnight culture was seeded into baffled flasks (125 ml) containing M9 medium (20 ml) plus 10 g/l sodium Glu (VeDan Co., Taiwan) unless stated otherwise. The composition of M9 medium comprised 6 g/l Na_2_HPO_4_, 3 g/l KH_2_PO_4_, 0.5 g/l NaCl, 1 g/l NH_4_Cl, 1 mM MgSO_4_, and 0.1 mM CaCl_2_. In the minimal medium, the dosage of Amp or of Kan was cut by half for use whenever necessary. The cell growth was followed by measuring the absorbance at 550 nm (OD_550_) with a spectrophotometer. The bacterial culture was conducted at 37°C with vigorously shaking. The protein production in the strain was induced by the addition of IPTG (1 mM) or L-arabinose (30 μM) upon the cell density reaching around 0.3 at OD_550_, followed by reducing the temperature to 30°C throughout the experiment. The analysis of Glu and organic acids essentially followed the reported method (Chiang et al., 2021a). In brief, Glu in the sample was derivatized by the addition of the solution containing borate buffer (pH 10.2), o-phthaldialdehyde solution, and 9-fluorenylmethoxycarbonyl chloride. The analysis was conducted using the ODS Hypersil column with HPLC Chromaster 5,160 (Hitachi, Tokyo, Japan). The mobile phase consisted of solution A (NaH_2_PO_4_‧H_2_O) and solution B (acetonitrile, methanol, and deionized water). Organic acids were analyzed using the Aminex HPX-87H column with the mobile phase containing 0.005 N H_2_SO_4_.

### Protein analysis

The bacterial culture (1 ml) was centrifuged at the end of experiments, and cell pellets were re-suspended in 0.2 ml Tris–HCl buffer (0.1 M) at pH 8.0. Cells were disrupted by sonication at the frequency of 20 kHz for 90 s (15 s of sonication followed by 15 s of pause). Cell-free extract (CFX) was obtained by recovering the supernatant after the cell lysate was centrifuged for 10 min at 12,000 × *g* at 4°C. The protein content of CFX was determined by Bio-Rad Protein Assay Kit. Proteins in CFX (20 μl) were analyzed by sodium dodecyl sulfate-polyacrylamide gel electrophoresis (SDS-PAGE). The HDT content as resolved in SDS-PAGE was quantified by using Image Analyzer GAS9000 (UVItech). The activity assay of HDT was determined according to the previous report (Chiang et al., 2014). In brief, the reaction was initiated at 40°C upon the addition of 10 μl CFX into the reaction solution (1 ml) consisting of 0.1 M Tris–HCl buffer (pH 8.0), 6 mM D,L-hydroxyphenyl hydantoin (HPH), and 0.5 mM MnCl_2_. The solution was heated at 100°C for 10 min to terminate the reaction which proceeded for 15 min. The remaining HPH was analyzed by HPLC and calculated for the volumetric activity (U/l) of HDT. The volumetric activity was then multiplied by the culture volume to give the total HDT yield (U).

## Results

### Protein production using Glu

**Figure 1:**
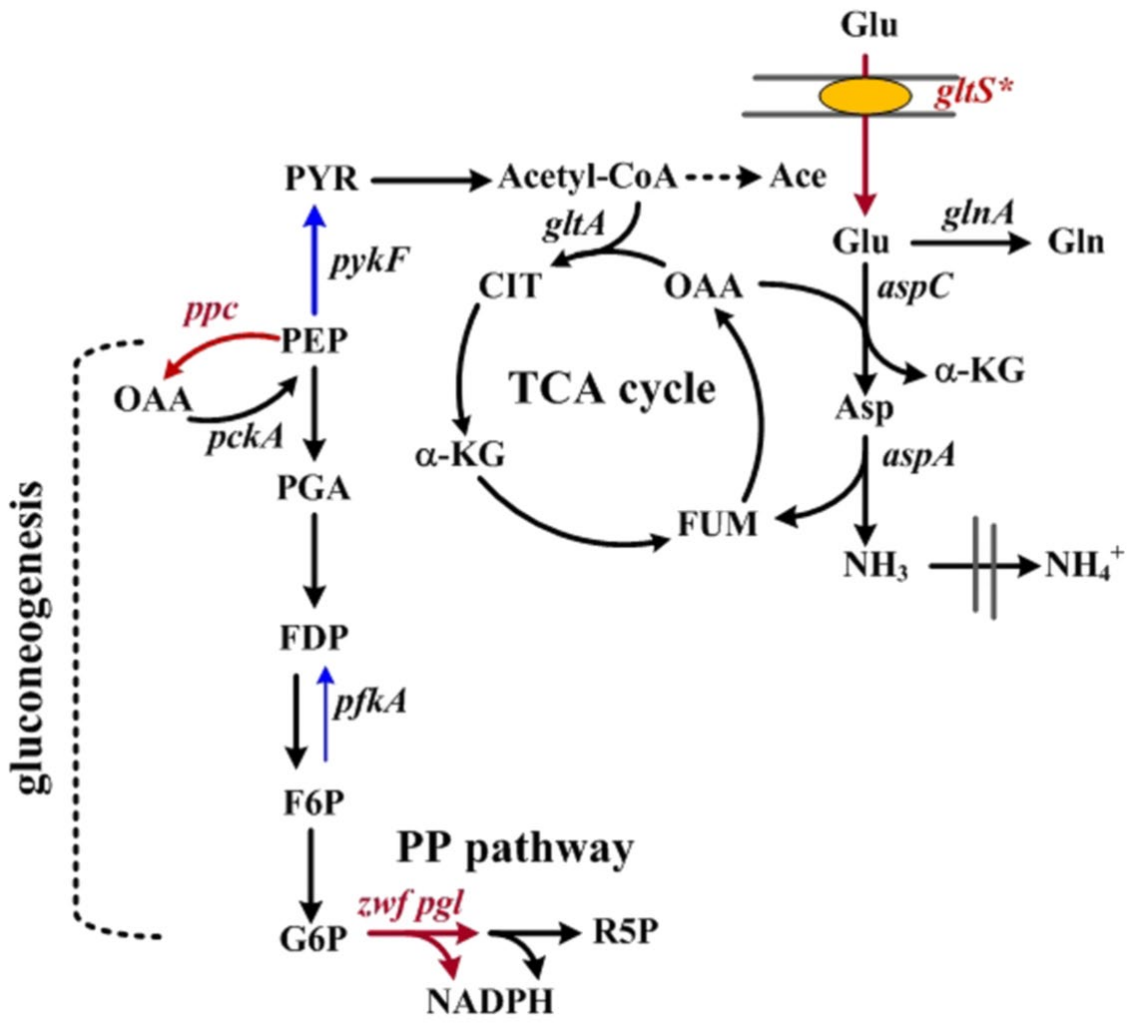
Schematic illustration of Glu metabolism in *Escherichia coli*. Related genes in metabolic pathways involve as follows: *aspC*, aspartate aminotransferase; *aspA*, Asp ammonia-lyase; *glnA*, glutamine synthetase; *gltA*, citrate synthase; gltS*, Na^+^/Glu symporter; *pckA*, phosphoenolpyruvate carboxykinases; *pfkA*, 6-phosphofructokinase; *pgl*, lactonase; *ppc*, PEP carboxylase; *pykF*, pyruvate kinase; *zwf*, glucose-6-phosphate dehydrogenase. Abbreviations: Ace, acetate; Asp, aspartate; CIT, citrate; F6P, fructose-6-phosphate; FDP, fructose-biphosphate; FUM, fumarate; G6P, glucose-6-phosphate; Gln, glutamine; α-KG, α-ketoglutarate; MAL, malate; OAA, oxaloacetate; PEP, phosphoenolpyruvate; PGA, 3-phosphoglyceraldehyde; PYR, pyruvate; R5P, ribose-5-phosphate.

HDT (E.C.3.5.2.2) from *Agrobacterium radiobacter* NRRL B11291 has an industrial application for manufacturing of unnatural D-amino acids and 3-carbamoyl-α-picolinic acid for the synthesis of antibiotics and modern agrochemicals like nicotinoid insecticides, respectively (Moriya et al., 1993). Without interfering with cell physiology, this enzyme was chosen as the model protein. The HDT production was first investigated in BL21(DE3) strain that harbored pET-TrChHDT plasmid containing P_T7_::HDT (Chiang et al., 2020b). The strain is known to carry the *lac*-dependent T7 expression system (Wagner et al., 2008). In addition, *E. coli* is unable to utilize Glu as a sole carbon source. This issue was addressed by construction of pTH-gltS* plasmid which carries gltS* with its own promoter. The physiological function of gltS* is responsible for the efficient transport of Glu (Figure 1), which renders *E. coli* capable of metabolizing Glu (Chiang et al., 2021b). Finally, the Glu-positive strain was obtained by introduction of pTH-gltS* plasmid into BL21(DE3) strain bearing pET-TrChHDT.

**Figure 2:**
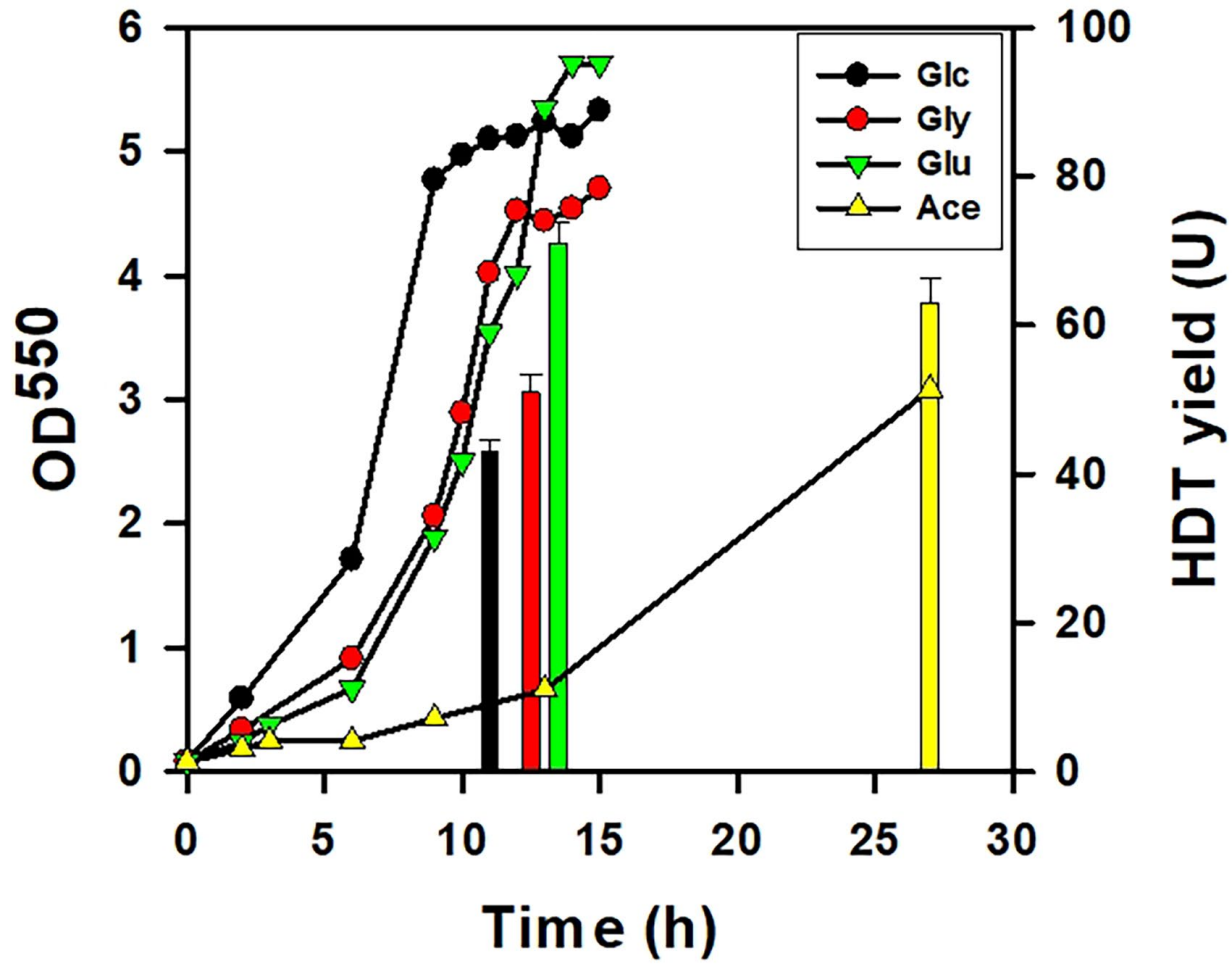
The protein production for the strain using various substrates. The HDT production was conducted with BL21(DE3) strain which harbored pET-TrChHDT plasmid in the presence of various carbon sources (10 g/l). The growth of the acetate-grown strain was sustained by supplement of yeast extract (2 g/l). To grow on Glu, the strain was additionally transformed with pTH-gltS* plasmid. A typical growth profile on each substrate and the HDT production (bars) were shown for the recombinant strain. HDT was analyzed for the strain grown on glucose (Glc), glycerol (Gly), Glu, and acetate (Ace) at 11 h, 13 h, 13 h, and 27 h, respectively. The experiment was performed in triplicate and data were shown with the standard deviation.

The HDT production in the strain was carried out with the minimal medium containing Glu, glucose, glycerol, or acetate. As shown in Figure 2, the cell growth was relatively well on the medium containing glucose, glycerol, and Glu. In sharp contrast to others, the strain exhibited poor growth on acetate. The total HDT production in terms of the enzyme activity (U) was determined for the strain grown on various carbon sources except acetate when entering the stationary growth phase. Interestingly, the Glu-grown strain produced the maximum level of HDT. It indicates that the protein production is promising for the strain dependent on Glu.

### The Glu-based production system

**Figure 3:**
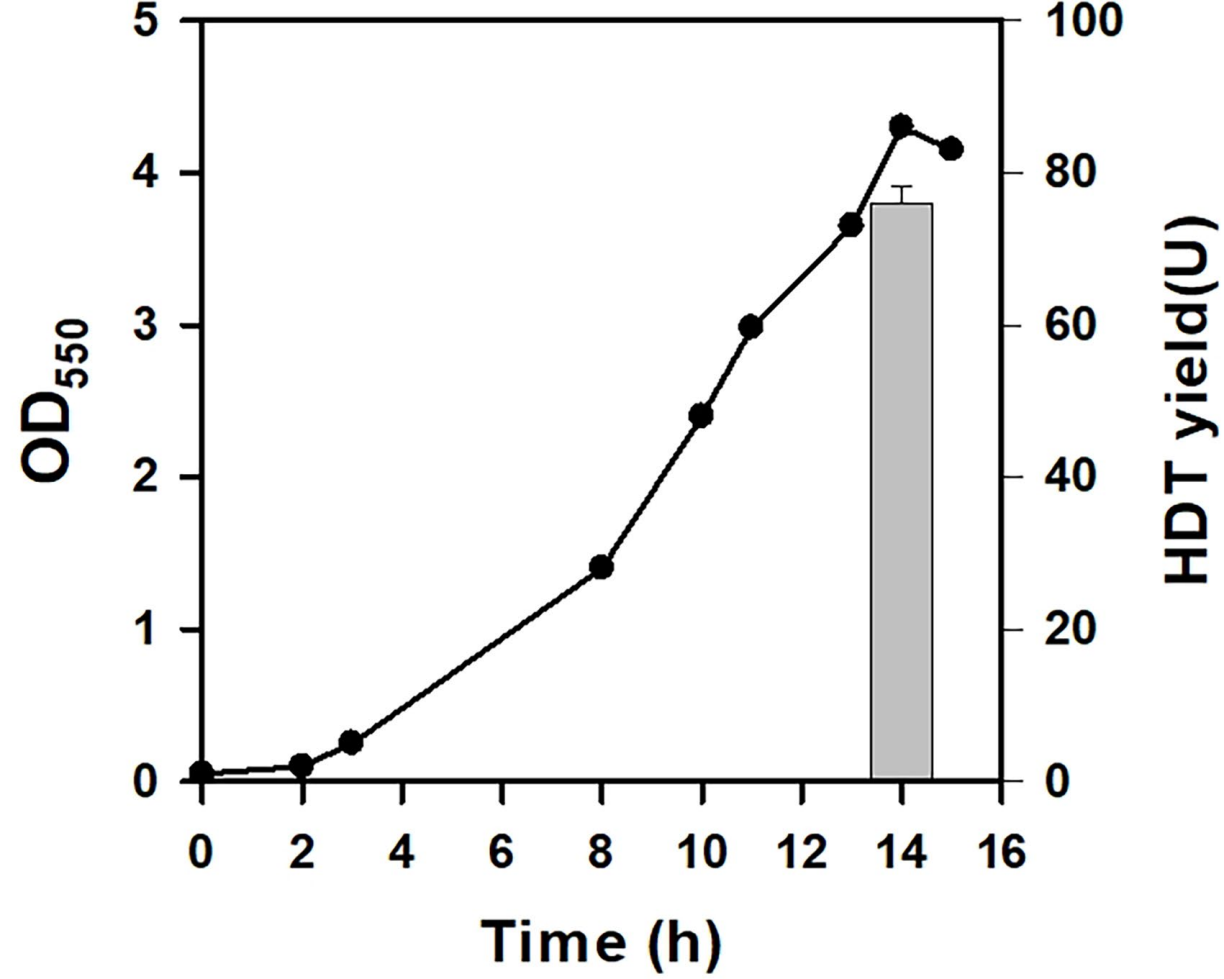
The protein production in Glu-dependent strain. The HDT production was conducted with BAD-HDT/pTH-gltS* strain in the presence of Glu (10 g/l). A typical growth profile and the HDT production (bar) were shown for the recombinant strain. The experiment was performed in triplicate and data were shown with the standard deviation.

BAD-5 strain harbors a genomic copy of L-arabinose (Ara)-inducible T7 expression system (Wang et al., 2011). Its Ara metabolism is interrupted by inactivation of *araBAD* operon, which, in turn, maintains the consistent inducibility of Ara. The Ara-based expression system has been illustrated to achieve the homogenous and efficient production of the cloned protein at a sub-saturated level of Ara. It was intriguing to investigate the usefulness of this expression system for the Glu-based protein production. To do so, BAD-5 strain was engineered by genomic insertion of P_T7_::HDT, consequently giving BAD-HDT strain. With pTH-gltS* plasmid, BAD-HDT strain was applied for the HDT production using Glu. As depicted in Figure 3, the strain (BAD-HDT/pTH-gltS*) produced HDT with a yield reaching 76 U. This production yield is comparable to that obtained by BL21(DE3) strain harboring the HDT-borne plasmid (Figure 2). Accordingly, BAD-HDT strain was employed for further investigations.

### Strain improvement

**Figure 4:**
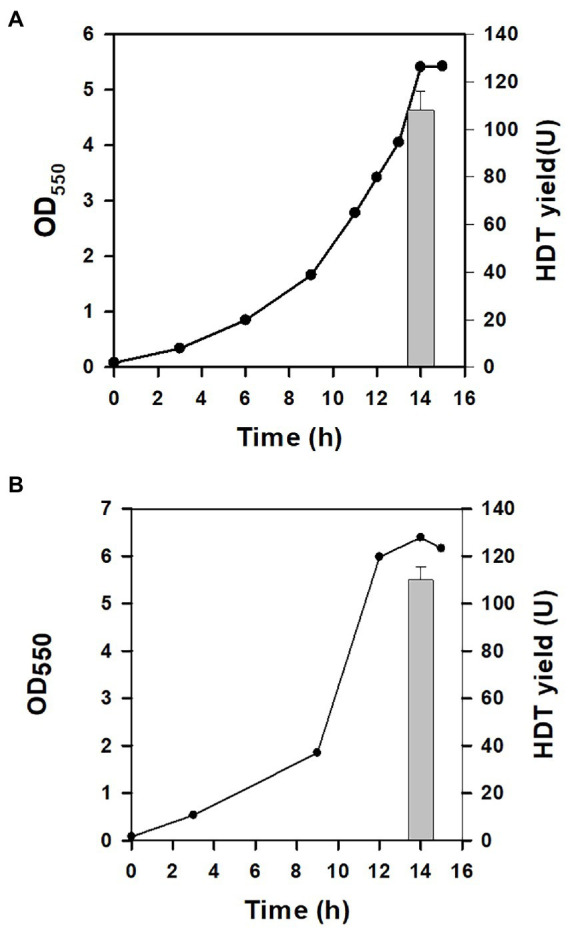
The protein production for the genetically-modified strain. The HDT production was conducted with BAD-HDT-1/pTH-gltS* strain **(A)** and BAD-HDT-1/pTH-gltS* strain bearing pACYC-aspA plasmid in the presence of Glu (10 g/l) **(B)**. A typical growth profile and the HDT production (bar) were shown for the recombinant strain. The experiment was performed in triplicate and data were shown with the standard deviation.

In our previous study, central metabolic pathways that affect the cell growth were identified in Glu metabolism involving the assimilation pathway, the tricarboxylic (TCA) cycle, and gluconeogenesis (Chiang et al., 2021b). In particular, gluconeogenesis links to the pentose phosphate (PP) pathway (Figure 1). The oxidative route of the PP pathway provides NADPH which is favorable for biosynthesis (White, 2007). We previously showed that the enhanced level of *zwf* (encoding glucose 6-phosphate dehydrogenase) and *pgl* (encoding lactonase) increases the intracellular level of NADPH (Saini et al., 2016). BAD-HDT strain was then engineered to overexpress *zwf* and *pgl* according to the reported method, resulting in BAD-HDT-1 strain. In the presence of Glu, the strain with pTH-gltS* plasmid (BAD-HDT-1/pTH-gltS*) enabled production of HDT at 108 U (Figure 4A).

In general, the flux distribution between the TCA cycle and gluconeogenesis allocates precursor metabolites available for biosynthesis of amino acids in Glu metabolism. The assimilation route consists of two consecutive reaction steps catalyzed by aspartate (Asp) aminotransferase (encoded by *aspC*) and Asp-ammonia lyase (encoded by *aspA*), which converts Glu to α-ketoglutarate (α-KG) in the TCA cycle. The activity of Asp-ammonia lyase was found to modulate the flux distribution in favor of the TCA cycle (Chiang et al., 2021b). Accordingly, BAD-HDT-1/pTH-gltS* strain was equipped with the T7A1 promoter-driven *aspA* by recruitment of pACYC-aspA plasmid (Wang et al., 2006). It resulted in a marginal increase in the HDT production for the resulting strain (Figure 4B).

### Improvement of the protein production

**Figure 5:**
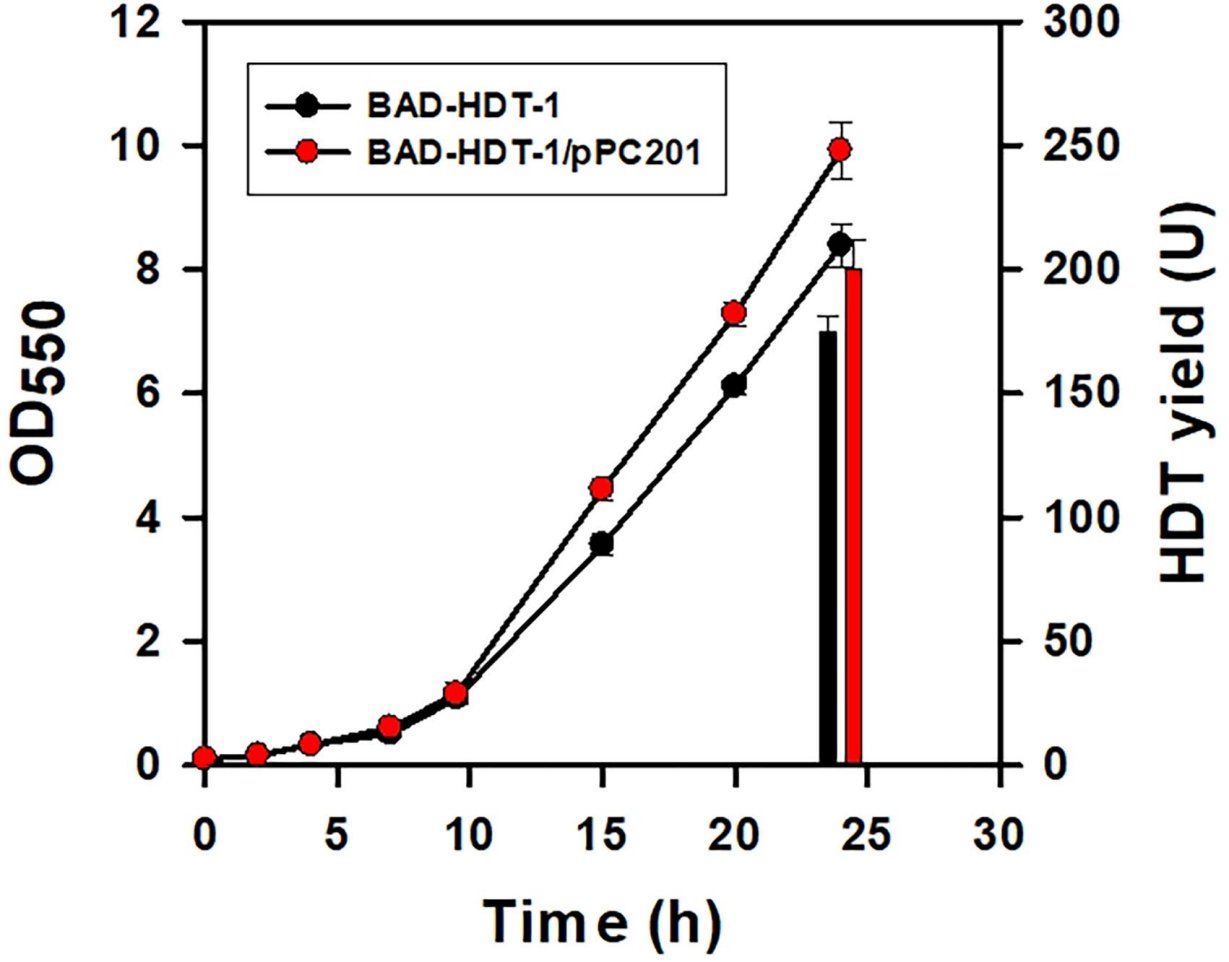
Improvement of the protein production in the engineered strain. The HDT production in BAD-HDT-1/pTH-gltS* and BAD-HDT-1/pTH-gltS*/pPC201 strain was carried out in the minimal medium containing Glu (20 g/l) and NH_4_Cl (0.35 g/l). A typical growth profile and the HDT production (bars) were shown for the recombinant strains. The experiment was performed in triplicate and data were shown with the standard deviation. Strains were designated as BAD-HDT-1 and BAD-HDT-1/pPC201 for clarity.

The deamination reaction involved in the assimilation pathway of Glu produces ammonium (Figure 1). The released ammonium may provide the nitrogen source for the Glu-dependent strain. To test this hypothesis, BAD-HDT-1/pTH-gltS* strain was cultured with the minimal medium containing Glu (10 g/l) and various levels of NH_4_Cl. The final biomass was determined after the cell grew at 14 h. Consequently, the cell density of the strain receiving the 1/3 dosage of NH_4_Cl remained unaffected as compared to that of the counterpart with a full dosage of NH_4_Cl. However, it caused a 10% decrease in the biomass when the 1/5 dosage of NH_4_Cl was administrated. The HDT production in BAD-HDT-1/pTH-gltS* strain was then conducted with the medium containing a 1/3 dosage of NH_4_Cl. In addition, the Glu level in the medium was doubled to increase the protein production. As shown in Figure 5, the HDT production in the strain reached 175 U.

It was found that BAD-HDT-1/pTH-gltS* strain produced a detectable level of acetate (*ca.* 1.3 g/l) in the presence of high Glu. The availability of phosphoenolpyruvate (PEP) leads to the formation of acetate from pyruvate (PYR) and is likely reduced by PEP carboxylase (encoded by *ppc*) which converts PEP to oxaloacetate (OAA) (Figure 1). BAD-HDT-1/pTH-gltS* strain was then transformed with pPC201 plasmid which contains the *trc* promoter-regulated *ppc* (Chao and Liao, 1993). Consequently, the resulting strain (BAD-HDT-1/pTH-gltS*/pPC201) produced HDT with 200 U and reduced the acetate production to 0.6 g/l (Figure 5).

## Discussion

Recombinant proteins have been commonly produced in *E. coli* using glucose. However, the adverse event of acetate overflow occurs in response to surplus glucose. Glycerol is utilized less efficiently than glucose in *E. coli*. This, in turn, lowers the production of acetate as a result of the induced “carbon stress-based acetate recycling pathway” (Martínez-Gómez et al., 2012). The use of glycerol is particularly useful for overproduction of aggregate-prone proteins which link to the stress response (Strandberg and Enfors, 1991). Nevertheless, an increase in glycerol utilization leads to PYR overflow in *E. coli* (Chiang et al., 2020a). Acetate is inexpensive but metabolized very slowly by *E. coli*. The acetate-based production of the single chain derivative of monellin was proven promising (Leone et al., 2015). However, the nutrient supplement is necessary to sustain growth of the strain dependent on acetate.

In this study, Glu was explored for production of the recombinant protein. BL21(DE3) strain is recognized as the most commonly used producer in the field. The recruitment of gltS* rendered the strain capable of utilizing Glu, which likely eliminates the kinetic constraint of Glu assimilation enzymes. The efficiency of the protein production was first evaluated in the strain using various substrates. Figure 2 showed that the maximum production of HDT was obtained for the strain grown on Glu. The strain metabolized various carbon sources at a different rates and displayed distinct growth profiles. Accordingly, the production rate (U/h) was calculated by dividing the HDT yield (U) by the time (h) in which the protein was sampled for the measurement. This gave the HDT productivity based on Glu, glycerol, glucose, and acetate reaching 5.5, 4.6, 3.9, and 2.3 U/h, respectively. Overall, Glu apparently serves as an excellent substrate for the protein production in *E. coli*.

The Glu-based production system was refined by development of BAD-HDT strain that carried the Ara-inducible T7 expression system and a genomic copy of P_T7_::HDT. In Glu metabolism, the assimilation pathway leads to the production of α-KG (Figure 1). The continued oxidation of α-KG in the TCA cycle produces OAA. PEP carboxykinase (encoded by *pckA*) decarboxylates OAA to form PEP, which mainly serves for gluconeogenesis. It is apparent that Glu metabolism closely relies on the TCA cycle. Notice that BAD-HDT strain belongs to *E. coli* B strain which displays a higher activity of the TCA cycle than K12 strain (Waegeman et al., 2011). To improve the protein production in the strain, the gluconeogenic flux was directed into the oxidative route in the PP pathway by enhanced expression of *zwf* and *pgl* (Figure 1). It resulted in a 42% increase in the HDT production for BAD-HDT-1/pTH-gltS* strain (Figure 4; Table 1). PYR kinase (encoded by *pykF*) is subjected to the repression of Cra (encoding fructose repressor) and controls the flow direction of glycolytic flux that bifurcates at the PEP node (Bledig et al., 1996). Cra is inactivated upon binding to intermediate metabolites (i.e., glycolytic intermediates) derived from glucose (Bley et al., 2018). Our previous study illustrated that the Glu-positive strain deprived of *pfkA* [encoding 6-phosphofructokinase (PFK)] failed to grow on Glu (Chiang et al., 2021b). The function of PFK is responsible for phosphorylation of fructose 6-phosphate to form fructose-1,6-biphosphate. The essential role of PFK in Glu metabolism likely elevates the level of glycolytic intermediates. This, in turn, disables Cra and induces the catabolite activation of *pykF*. Accordingly, the further oxidization of PEP mediated by PYR kinase may restrict the gluconeogenic flux (Figure 1). This flux constraint appears to be eased by activation of the oxidative PP pathway after recruitment of *zwf* and *pgl*.

**Table 1:** The summary of engineered strains applied for the HDT production.

Strain	μ (1/h)	X (g DCW/l)	Y (U/g DCW/l)
BAD-HDT/pTH-gltS*	0.25 ± 0.01	1.42 ± 0.06	53.5
BAD-HDT-1/pTH-gltS*	0.27 ± 0.02	1.82 ± 0.06	59.3
BAD-HDT-1/pTH-gltS*/pPC201	0.23 ± 0.01	3.30 ± 0.08	60.6

Engineered strains were applied for the HDT production based on Glu. Their specific growth rate (μ), final biomass (*X*), and specific HDT yield (*Y*) were summarized in the Table. DCW, dry cell weight.

The reaction catalyzed by Asp-ammonia lyase releases ammonia in Glu metabolism (Figure 1). It was illustrated that BAD-HDT-1/pTH-gltS* strain displayed no growth defect in the Glu-containing medium with a 1/3 dosage of NH_4_Cl. The result suggests that the liberated ammonium supplements the nitrogen source which is sufficient to sustain cell activities. In contrast, the administration of a 1/3 dosage of NH_4_Cl caused a decrease (>50%) in the biomass of the strain grown on glucose, glycerol, or acetate. The HDT production in BAD-HDT-1/pTH-gltS* strain was further improved in the presence of high Glu (*ca.* 20 g/l) and low NH_4_Cl (*ca.* 0.33 g/l) (Figure 5). However, the approach resulted in the accumulation of acetate with a concentration lower than the inhibitory level. The underlying mechanism for acetate overflow remains unclear. With an enhanced level of *ppc*, BAD-HDT-1/pTH-gltS*/pPC201 strain enabled reduction of acetate and production of 1.63-fold more HDT than its parent strain (BAD-HDT/pTH-gltS*). It is likely that the activity of PEP carboxylase reduces the PEP pool and provides more OAA in favor of the reaction catalyzed by citrate synthase (encoded by *gltA*) (Figure 1). Notice that the availability of Glu leads to formation of glutamine (Gln) through Gln synthetase (encoded by *glnA*). The reductive synthesis of more than 10 amino acids requires ammonia supplied by Glu and Gln (White, 2007). This apparently adds one more advantage to Glu applied for the protein production.

In summary, this preliminary study illustrated the potential of Glu for overexpressing recombinant proteins in *E. coli*. Glu is the first commercially-available amino acid as produced by the fermentation process, and its market size reaches 1.5 million tons per year and increasingly grows (Kumar et al., 2014). In addition, the abundance of Glu exists in the protein waste associated with the conventional biomass-treatment process. It is expected that waste streams of these production processes would provide a sustainable and renewable source of Glu. The focus of our continued work is development of a streamlined process to recover Glu from the protein waste. Accordingly, the developed approach based on Glu may open up a new avenue for the enzyme biorefinery platform.

## Data availability statement

The raw data supporting the conclusions of this article will be made available by the authors, without undue reservation.

## Author contributions

C-JC and Y-PC conceived the idea and wrote the manuscript. M-CH and TT performed the experiment. All authors contributed to the article and approved the submitted version.

## Funding

This work was co-supported by China Medical University (CMU111-MF-79) and the Ministry of Science and Technology (MOST 108-2221-E-035-052-MY3), Taiwan.

## Conflict of interest

The authors declare that the research was conducted in the absence of any commercial or financial relationships that could be construed as a potential conflict of interest.

## Publisher’s note

All claims expressed in this article are solely those of the authors and do not necessarily represent those of their affiliated organizations, or those of the publisher, the editors and the reviewers. Any product that may be evaluated in this article, or claim that may be made by its manufacturer, is not guaranteed or endorsed by the publisher.
